# The Utilization of Glucagon-like Peptide 1 Agonists and Risk of Following External Eye Diseases in Type 2 Diabetes Mellitus Individuals: A Population-Based Study

**DOI:** 10.3390/healthcare11202749

**Published:** 2023-10-17

**Authors:** Ying-Chi Fan, Shu-Yen Peng, Chao-Kai Chang, Chia-Yi Lee, Jing-Yang Huang, Ming-Ju Hsieh, Shun-Fa Yang

**Affiliations:** 1 Institute of Medicine, Chung Shan Medical University, Taichung 402, Taiwan; 2 Department of Neurology, Chung Shan Medical University Hospital, Taichung 402, Taiwan; 3 School of Medicine, Chung Shan Medical University, Taichung 402, Taiwan; 4 Department of Ophthalmology, Jen-Ai Hospital Dali Branch, Taichung 412, Taiwan; 5 Nobel Eye Institute, Taipei 100, Taiwan; 6 Department of Optometry, Da-Yeh University, Chunghua 515, Taiwan; 7 Department of Medical Research, Chung Shan Medical University Hospital, Taichung 402, Taiwan; 8 Oral Cancer Research Center, Changhua Christian Hospital, Changhua 500, Taiwan; 9 Department of Post-Baccalaureate Medicine, College of Medicine, National Chung Hsing University, Taichung 402, Taiwan; 10 Graduate Institute of Biomedical Sciences, China Medical University, Taichung 404, Taiwan

**Keywords:** GLP-1 agonists, dry eye disease, superficial keratitis, epidemiology, age

## Abstract

The glucagon-like peptide 1 (GLP-1) agonist showed anti-hyperglycemic and anti-inflammatory effects, which may retard the risk of external eye disease. The protective effect of GLP-1 agonist and dry eye disease (DED) was found, while the relationship between GLP-1 agonist and other corneal diseases was not clear. Herein, we aim to evaluate the association between the usage of GLP-1 agonists and the development of the following external eye disease in type 2 diabetes mellitus (T2DM) patients. A retrospective cohort study using the National Health Insurance Research Database (NHIRD) of Taiwan was conducted. The T2DM patients were divided into those with GLP-1 treatment and those without GLP-1 treatment and matched with a 1:2 ratio. The main outcomes were the development of dry eye disease (DED), superficial keratitis, and infectious keratitis. The Cox proportional hazard regression was adopted to produce the adjusted hazard ratio (aHR) with a 95% confidence interval (CI) of external eye diseases between groups. There were 115, 54, and 11 episodes of DED, superficial keratitis, and infectious keratitis in the GLP-1 group. Another 280, 168, and 31 events of DED, superficial keratitis, and infectious keratitis were recorded in the control group. The GLP-1 group demonstrated a significantly lower incidence of DED (aHR: 0.853, 95% CI: 0.668–0.989, *p* = 0.0356) and superficial keratitis (aHR: 0.670, 95% CI: 0.475–0.945, *p* = 0.0107) compared to the control group. In the subgroup analyses, the correlation of GLP-1 agonist and DED development was more prominent in patients younger than 60 years old (*p* = 0.0018). In conclusion, the GLP-1 agonist treatments are significantly associated with a lower incidence of subsequent DED and superficial keratitis, while the relationship was not significant between GLP-1 agonist usage and infectious keratitis.

## 1. Introduction

Type 2 diabetes mellitus (T2DM) refers to the hyperglycemic status, which results from insulin resistance [1]. Advanced T2DM can cause prominent morbidities like cerebrovascular accidents, acute myocardial infarction, and some ocular surface diseases [2,3]. Concerning the treatment of T2DM, the main therapy is the usage of anti-diabetic medication and insulin injections for severe cases [4]. Among the anti-diabetic medications, glucagon-like peptide 1 (GLP-1) agonists demonstrated a 2 percent decrement in glycated hemoglobin in a previous study [5].

The GLP-1 showed a protective effect other than glycemic control in previous publications [5]. The treatment with GLP-1 agonists can preserve kidney function in general and T2DM patients [6,7]. On the other hand, the treatment with GLP-1 agonists decreased the rate of major adverse cardiovascular events and obesity in earlier studies [8,9]. In addition, the GLP-1 agonists can reduce the inflammation response by suppressing macrophage aggregation and interleukin-6 expression [10], and the decrement of blood glucose levels can also reduce the inflammation, which can be achieved by the GLP-1 agonist treatments [11]. Accordingly, the GLP-1 agonists may influence the formation of other inflammatory diseases.

Previous literature has discussed the association between the treatment with GLP-1 agonist and the eye [12,13,14]. The GLP-1 agonist treatments had no effect on the severity of diabetic retinopathy [14], while other studies showed that the GLP-1 agonist treatment is associated with an increased risk (odds ratio: 1.23) of rapidly worsening diabetic retinopathy [13]. This phenomenon may have resulted from the elevation of vascular endothelial growth factor levels and angiogenesis in patients who received GLP-1 agonist treatment [15]. Moreover, the usage of metformin, sodium-glucose cotransporter-2 inhibitors, GLP-1 agonists, or combined anti-diabetic therapy is associated with lower dry eye disease (DED) incidence [16,17,18]. Still, there is no known research discussing the relationship between GLP-1 agonist treatment and external eye diseases like keratopathy. Since the GLP-1 agonist can suppress inflammation, which is the main mechanism of DED and keratopathy [7,19,20], the protective effect of the GLP-1 agonist on DED and keratopathy may be possible. 

In epidemiological research, T2DM is a widely spread metabolic disease throughout the world. The annual prevalence of T2DM is about 8.8 percent, which may increase to 9.9 percent in the year 2040 [21]. Among them, several types of GLP-1 agonists have been applied with acceptable therapeutic outcomes [22]. On the other hand, the prevalence of DED ranges from 5 to 50 percent throughout the world [23], and in prevalent areas like Japan, the prevalence of DED can reach 76.5 percent in women [24]. Among the DED population, many patients experience severe DED symptoms, poor quality of life, and a considerable cost [23]. Moreover, the presence of DED is associated with the development of major depressive disorder [25]. Superficial keratitis and infectious keratitis, although not as prevalent as DED, also have an incidence of 2.5–27.6 per 100,000 population per year in the US [26]. Since the GLP-1 agonist is a common medication and external eye disease can lead to significant visual impairment and reduced quality of life, the protective effect of the GLP-1 agonist on external eye disease development could be illustrated.

The purpose of this study is to evaluate the possible relationship between the treatment with GLP-1 agonist and the incidence of external eye diseases, including DED, superficial keratitis, and infectious keratitis. The current study used data from the Taiwan National Health Insurance Research Database (NHIRD).

## 2. Materials and Methods

### 2.1. Data Source

The method in the present study adhered to the Declaration of Helsinki in 1964, and its late amendments and the present study was approved by both the National Health Insurance Administration of Taiwan and the Institutional Review Board of Chung Shan Medical University (Project code: CS1-20108). The necessity of written informed consent was waived by the above two institutions. The Taiwan NHIRD preserves the claimed data of nearly 23 million citizens of Taiwan with a time interval from 1 January 2014 to 31 December 2020. Compared to the real medical records that present the disease course, exam results, and therapeutic outcome, the claimed data only illustrated the basic demography, diagnostic codes, exam codes, and the types of medication/management patients received. In other words, only the existence of a code or not, rather than the value of examination or prognosis after treatment, is available in the claimed data. The available data in the Taiwan NHIRD includes the International Classification of Diseases-Ninth Revision (ICD-9), the International Classification of Diseases-Tenth Revision (ICD-10) diagnostic codes, age, sex, income level, education level, urbanization level, occupation, medical department codes, image exam codes, laboratory exam codes, surgery codes, procedure codes, and the international ATC codes for pharmaceutical management.

### 2.2. Patient Selection

We conducted a retrospective cohort study, and individuals were defined as T2DM with GLP-1 agonist usage if they achieved these inclusion criteria: (1) the receipt of T2DM diagnosis via related ICD-9 or ICD-10 codes from 2014 to 2019, (2) had visited family medicine or internal medicine administration with a follow-up period longer than three months via department codes, and (3) the GLP-1 agonist applications including exenatide and liraglutide via the international ATC codes. The common forms and dosages of GLP-1 agonists in Taiwan were listed as follows: exenatide (5 mcg, twice per day), exenatide LAR (2 mg, once a week), and liraglutide (0.6 mg, once per day). The index date of our study was set as a date 6 months after the starting of GLP-1 agonist therapy. In addition, the following exclusion criteria were applied to standardize our study population: (1) no demographic data, (2) application of GLP-1 agonist before the T2DM diagnosis, (3) the presence of DED, superficial keratitis, and infectious keratitis before the index date, and (4) the usage of GLP-1 agonist was less than 6 times. After that, each T2DM patient using a GLP-1 agonist was matched to two T2DM patients who did not use a GLP-1 agonist, and the latter population constituted the control group. We used the 1:2 ratio since it can enroll adequate patient numbers without a significant imbalance of case numbers between the two groups. In addition, we used G-power software (version 3.1.9.2 Heinrich-Heine-Universität Düsseldorf, Germany) to calculate the statistical power of our study. The statistical test was “correlation: Point biserial model”, the type of power analysis was a compromise, the sample size was set as 1000 in both groups, and the effect size was 0.5. The alpha value and statistical power (1 minor beta) of our study were 0.01 and 0.99, respectively, due to the large population, which included more than 1000 individuals. The propensity score-matching (PSM) method was used in our study to match the two groups by adjusting the demographic data, systemic disease factors, and medical factors. After the whole selection procedure, a total of 2007 and 4014 T2DM patients were included in the GLP-1 group and the control group.

### 2.3. Main Outcome

The main outcome in this study was newly-developed external eye disease events via the following criteria: (1) the presence of DED, superficial keratitis, and infectious keratitis diagnosis according to related ICD-9 and ICD-10 diagnostic codes, (2) the applications of slit-lamp biomicroscopy examination before or at the same time of external eye disease diagnoses via the procedure codes, (3) the treatment with topical antibiotic eyedrops or artificial tears after the external eye disease diagnoses by the ATC codes, (4) the external eye disease diagnoses were made by an ophthalmologist. Patients in the present study were traced until the accomplishment of the following conditions: (1) external eye disease diagnoses, (2) patient withdrawn from our National Health Insurance program, or (3) the deadline of NHIRD, which indicates 31 December 2020.

### 2.4. Predisposing Factors

In the present study, we included the effect of certain demographic data, co-morbidities, and medications in the statistical analysis to adjust the effect of predisposing factors on external eye disease development: age, sex, economic level, hypertension, hyperlipidemia, ischemic heart diseases, rheumatoid arthritis, systemic lupus erythematosus, Sjogren syndrome, ankylosing spondylitis, biguanides, sulfonylureas, alpha-glucosidase inhibitors, thiazolidinediones, dipeptidyl peptidase-4 inhibitor, calcium channel blockers, diuretics, statin, and benzodiazepines according to the ICD-9 diagnostic codes, ICD-10 diagnostic codes, and ATC codes in the NHIRD. We adjusted the systemic inflammatory diseases mentioned above since they could be possible risk factors for the development of DED, which is also an inflammatory disease [20]. To ensure the durations of these predisposing factors are long enough to influence the risk of external eye disease development, only the factors with an interval longer than two years before the index date were enrolled in statistical analyses.

### 2.5. Statistical Analysis

The SAS version 9.4 (SAS Institute Inc, Cary, NC, USA) was used in our statistical analyses. Descriptive analyses were exploited to show the demography, co-morbidities, and co-medications between the two groups, and the absolute standardized difference (ASD) was introduced to analyze the difference between the GLP-1 and control groups. An ASD value higher than 0.1 was defined as statistical significance between the two groups. In the next step, the Cox proportional hazard regression was done to provide the adjusted hazard ratios (aHR) with associated 95% confidence intervals (CI) of external eye disease incidence between the GLP-1 and control groups and the effect of demographic features, co-morbidities, and co-medications were adjusted in the Cox proportional hazard regression. In the subgroup analyses, the T2DM patients were stratified by age and sex, and then Cox proportional hazard regression was done again to analyze the aHR with 95% CI of external eye diseases in different subgroups. Moreover, the interaction test was done to demonstrate the influence of GLP-1 agonists on the external eye disease incidence in different subgroups. The statistical significance was depicted as *p* < 0.05 in this research, and a *p*-value lower than 0.0001 was illustrated as *p* < 0.0001.

## 3. Results

### 3.1. Demographic Characters of GLP-1 and Control Groups

**Figure 1:**
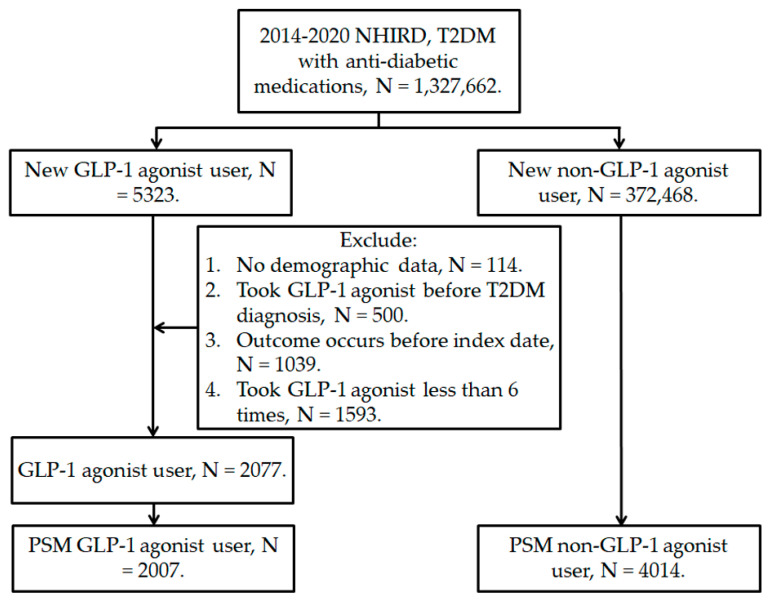
The flowchart of participant selection. NHIRD: National Health Insurance Research Database, N: number, T2DM: type 2 diabetes mellitus, GLP-1: glucagon-like peptide 1, PSM: propensity score-matching.

The flowchart of patient selection is presented in Figure 1. The clinical features of the GLP-1 and control groups are presented in Table 1. The sex distribution was identical between the two groups due to the PSM procedure. Also, the age and economic level distributions were also similar between the two groups (both ASD < 0.1). For the systemic diseases and medications, the GLP-1 group showed a higher rate of dipeptidyl peptidase-4 inhibitor treatment than the control group (ASD = 0.251), while the distributions of other factors were similar between the two groups (all ASD < 0.1) (Table 1).

### 3.2. Risk of External Eye Diseases in GLP-1 Population

After a follow-up period of up to 6 years, there were 115, 54, and 11 episodes of DED, superficial keratitis, and infectious keratitis in the GLP-1 group. Another 280, 168, and 31 events of DED, superficial keratitis, and infectious keratitis were recorded in the control group. According to the results of Cox proportional hazard regression, the GLP-1 group demonstrated a significantly lower incidence of DED (aHR: 0.853, 95% CI: 0.668–0.989, *p* = 0.0356) and superficial keratitis (aHR: 0.670, 95% CI: 0.475–0.945, *p* = 0.0107) compared to the control group (Table 2). However, the incidence of infectious keratitis was similar between the two groups (aHR: 0.597, 95% CI: 0.271–1.318, *p* = 0.5563) (Table 2).

### 3.3. Subgroup Analyses on GLP-1 Group

In the subgroup analyses, the aHR of DED was significantly lower in the GLP-1 group in both the populations aged younger or older than 60 years old (both the upper limits of 95% CI below 1). Furthermore, the association of GLP-1 agonist and DED development was more prominent in patients younger than 60 years old (*p* = 0.0018). The female population using GLP-1 showed a non-significant aHR of DED, but the difference between males and females was insignificant (*p* = 0.0647). On the other hand, the incidence of superficial keratitis and infectious keratitis were not affected by age or sex (all 95% CI included 1) (Table 3).

## 4. Discussion

Briefly, the present study demonstrated a lower incidence of DED and superficial keratitis in T2DM patients with GLP-1 agonist treatment than the non-GLP-1 usage T2DM individuals. Moreover, the lower incidence of DED was more significant in the T2DM patients taking GLP-1 agonists and aged younger than 60 years old. On the other side, the incidence of infectious keratitis was not affected by the treatment with GLP-1.

The GLP-1 agonist has several mechanisms to reduce the progression of T2DM and other morbidities [5]. The glucagon-like function of the GLP-1 agonist reduces blood sugar effectively in general T2DM patients [27]. In addition to the anti-hyperglycemia function, the GLP-1 agonist also demonstrated an anti-inflammatory effect in a previous study [28]. Another preceding study illustrated that the GLP-1 would be secreted if a high level of interleukin-6 and lipopolysaccharides was detected [29]. In addition, the GLP-1 agonist could retard the inflammation and glomerular alteration in diabetic kidney disease [30]. On the other hand, the oxidative stress would also be altered by the presence of GLP-1 agonist in which glomerular sclerosis and oxidative stress were decreased after the addition of GLP-1 agonist in experimental research [31]. The GLP-1 agonist is related to some neuro-protective benefits by the anti-apoptotic and anti-oxidative mechanisms [32]. 

DED is an inflammatory disease in recent research with higher interleukin and tumor necrosis factor expressions and higher tear osmolarity [20]. Certain anti-inflammatory medications like cyclosporine and rebamipide can reduce the severity of DED effectively [33]. In addition, the oxidative stress of the ocular surface would be involved in the formation of DED [33]. The inflammation was also found in the corneal lesions, like superficial punctate keratitis and infectious keratopathy [26,34], and the excess oxidative stress is associated with ocular surface damage [19]. Because DED and corneal diseases can be aggravated by inflammation and oxidative stress [19,20,34], we speculate that the addition of GLP-1 agonists can reduce the incidence of these external eye diseases due to their anti-inflammatory and antioxidant effects [22]. The concept was supported by the results of the present study.

In the present study, the treatment with GLP-1 agonist is associated with a lower incidence of DED and superficial keratitis. Few researchers have focused on the anterior segment of the eye, except for two studies that illustrated the protective effect of GLP-1 on the incidence of DED [16,17]. To our knowledge, the present study may be a novel experience to reveal the possible association between GLP-1 agonist treatment and the lower incidence of subsequent external eye disease, including corneal lesions. Also, the patients with a previous history of DED and corneal diseases were excluded from the present study, and some confounding factors for external eye diseases, including age, sex, and autoimmune diseases, were adjusted in the Cox proportional hazard regression model. Moreover, the T2DM patients using GLP-1 agonist less than six times were excluded from the present study to ensure the concentration and duration of GLP-1 agonist is adequate. Consequently, the GLP-1 agonist treatment may be an independent protective factor for DED and superficial keratitis. The GLP-1 agonist treatment can suppress both inflammation and oxidative stress by regulating the expression of tumor necrosis factor, matrix metalloproteinase, and reactive oxygen species [10]. The above three pathways are associated with the development of DED [19,20] and also lead to inflammation as well as oxidative stress in the experimental keratitis model [35]. In addition, the usage of other metabolic medications like sodium-glucose cotransporter-2 inhibitors can also reduce the incidence of external eye diseases like DED [16,17]. As a result, the GLP-1 agonist, which has a similar anti-inflammatory function to the sodium-glucose cotransporter-2 inhibitors, may also reduce the incidence of external eye disease. Since the major pathophysiology of infectious keratitis was microorganism invasion, it may be reasonable that the usage of GLP-1 agonist had no effect on the incidence of infectious keratitis.

In the subgroup analyses, the incidence of DED was lower in all age subgroups that used GLP-1 agonists. In addition, the correlation of GLP-1 agonist treatment and low DED incidence was more significant in those younger than 60 years old. There was seldom research to report this condition. Contrary to our study, old age is a well-known risk factor for the development of DED, in which individuals aged less than 45 years demonstrated a DED prevalence of only 2.7 percent in a study that enrolled 75,000 participants [23]. We think that old age may allow DED to occur more easily, and thus, the relationship between GLP-1 agonist treatment and lower DED incidence was weaker in the older population compared to their younger counterparts. On the other hand, the female population with T2DM and using GLP-1 agonists showed an insignificant association between GLP-1 agonist usage and DED incidence (all *p* > 0.05). Being female is another known risk factor for DED, where the odds ratio of DED is 2.6-fold in the female population compared to the male population from a study that enrolled 79,866 participants [36], which is also conflicting with the results of our study. This may be the cause of an insignificant relationship between GLP-1 agonist usage and DED incidences in women since they are more vulnerable to DED development. Concerning superficial keratitis and infectious keratitis, there was no difference in the association between GLP-1 agonist usage and disease development in all the subgroups. The phenomenon of our study may indicate that age and sex are not risk factors for the two external eye diseases.

There are still some limitations in the present study. Firstly, we used the claimed data rather than the real medical documents in the present study; thus, several important information like the initial level of blood sugar as well as glycated hemoglobin, the exact frequency of GLP-1 agonist treatment in each patient, the level of serum C-reactive protein, the level of blood sugar and glycated hemoglobin after the GLP-1 agonist treatment, the external eye photography of external eye diseases, the results of DED-related examinations, the culture results of infectious keratitis, the therapeutic outcome of external eye disease, the recurrence of external eye disease, and the detailed illness history of other diseases cannot be evaluated. Secondly, the retrospective design of the present study would decrease the homogeneity of the study population, although we applied PSM to standardize the general condition between the GLP-1 and control groups. In addition, the patients often used several anti-hyperglycemic medications, which may influence our results, although we adjusted the effect of all anti-hyperglycemic medications in our statistical analyses. Finally, nearly all the participants in the present study are Han Taiwanese; thus, the external validity of the present study was low.

## 5. Conclusions

In conclusion, the utilization of GLP-1 agonists is associated with a lower incidence of following DED and superficial keratitis after adjusting multiple confounders. Furthermore, the relationship between GLP-1 agonist treatment and reduced DED incidence was more significant in T2DM patients aged younger than 60 years. Consequently, T2DM individuals with known risk factors for DED or superficial keratitis may be suggested to take a GLP-1 agonist if there is no contraindication. Further large-scale studies to evaluate the effect of combined therapy of GLP-1 agonists and other anti-diabetic agents on the development of superficial keratitis and treatment outcomes of external eye diseases are mandatory.

## Figures and Tables

**Table 1:** The basic characteristics between the GLP-1 and non-GLP-1 groups.

Characters	Non-GLP1 (N = 4014)	GLP-1 (N = 2007)	ASD
Sex			0.0000
Male	2264 (56.40%)	1132 (56.40%)	
Female	1750 (43.60%)	875 (43.60%)	
Age			0.0032
20–39	1043 (25.98%)	625 (31.14%)	
40–49	1377 (34.30%)	658 (32.79%)	
50–59	1089 (27.13%)	486 (24.22%)	
60–69	420 (10.46%)	184 (9.17%)	
70–79	57 (1.42%)	42 (2.09%)	
≥80	28 (0.70%)	12 (0.60%)	
Economic level			0.0052
Low	1116 (27.80%)	585 (29.15%)	
Low-moderate	2003 (49.90%)	964 (48.03%)	
Moderate	572 (14.25%)	307 (15.30%)	
High	323 (8.05%)	151 (7.52%)	
Co-morbidity			
Hypertension	1746 (43.50%)	914 (45.54%)	0.0023
Ischemic heart diseases	197 (4.91%)	147 (7.32%)	0.0541
Hyperlipidemia	2385 (59.42%)	1346 (67.07%)	0.0364
Rheumatoid arthritis	14 (0.35%)	14 (0.70%)	0.0011
Systemic lupus erythematosus	7 (0.17%)	3 (0.15%)	0.0005
Sjogren syndrome	14 (0.35%)	6 (0.30%)	0.0006
Ankylosing spondylitis	38 (0.95%)	22 (1.10%)	0.0005
Co-medication			
Biguanides	3370 (83.96%)	1808 (90.08%)	0.0084
Sulfonylureas	1230 (30.64%)	869 (43.30%)	0.0416
Alpha-glucosidase inhibitors	146 (3.64%)	177 (8.82%)	0.0357
Thiazolidinediones	180 (4.48%)	200 (9.97%)	0.0187
Dipeptidyl peptidase 4 inhibitor	929 (23.14%)	1028 (51.22%)	0.1243
Calcium channel blockers	688 (17.14%)	311 (15.50%)	0.0025
Diuretics	966 (24.07%)	891 (44.39%)	0.0865
Statin	1620 (40.36%)	1111 (55.36%)	0.0326
Benzodiazepines	445 (11.09%)	204 (10.16%)	0.0058

GLP-1: glucagon-like peptide 1, N: number, ASD: absolute standard difference.

**Table 2:** The risk of external eye disease between the GLP-1 and non-GLP-1 groups.

Events	Non-GLP-1 Group	GLP-1 Group	*p* Value
DED			
Person-months	111,953	58,044	
Event	280	115	
Crude HR (95% CI)	Reference	0.795 (0.640–0.987)	
aHR (95% CI)	Reference	0.853 (0.668–0.989) *	0.0356 *
Superficial keratopathy			
Person-months	114,809	58,866	
Event	168	54	
Crude HR (95% CI)	Reference	0.628 (0.463–0.854)	
aHR (95% CI)	Reference	0.670 (0.475–0.945) *	0.0107 *
Infectious keratitis			
Person-months	117,866	59,855	
Event	31	11	
Crude HR (95% CI)	Reference	0.699 (0.351–1.391)	
aHR (95% CI)	Reference	0.597 (0.271–1.318)	0.5563

GLP-1: glucagon-like peptide 1, DED: dry eye disease, aHR: adjusted hazard ratio, CI: confidence interval. * denotes significant differences between the two groups.

**Table 3:** The subgroup analyses for corneal disease development stratified by age and sex.

Subgroup	aHR	95% CI	P for Interaction
DED			
Age			0.0018 *
<60	0.776	0.624–0.901	
≥60	0.878	0.687–0.997	
Sex			0.0647
Male	0.843	0.649–0.980	
Female	0.864	0.675–1.004	
Superficial keratopathy			
Age			0.3824
<60	0.665	0.483–0.926	
≥60	0.689	0.460–0.963	
Sex			0.1045
Male	0.681	0.492–0.951	
Female	0.666	0.450–0.933	
Infectious keratitis			
Age			0.2223
<60	0.621	0.255–1.467	
≥60	0.544	0.282–1.298	
Sex			0.2027
Male	0.633	0.292–1.410	
Female	0.576	0.264–1.300	

DED: dry eye disease, aHR: adjusted hazard ratio, CI: confidence interval. * denotes significant differences between the two groups.

## Data Availability

Due to the policy of the National Health Insurance Administration in Taiwan, the raw data of this study are not available.
